# Special Considerations for Measuring Energy Expenditure with Doubly Labeled Water under Atypical Conditions

**DOI:** 10.4172/2165-7904.S5-002

**Published:** 2015-07-30

**Authors:** Surabhi Bhutani, Natalie Racine, Tim Shriver, Dale A Schoeller

**Affiliations:** Department of Nutritional Sciences, University of Wisconsin-Madison, Wisconsin, 53706, USA

**Keywords:** Total energy expenditure, Doubly labeled water, Obesity

## Abstract

The global increase in the prevalence of obesity has dramatically increased interest in understanding the factors that influence human total energy expenditure (TEE). This in turn has increased interest in the doubly labeled water (DLW) method, a technique for measurement of total energy expenditure in free-living humans. The increasing use of this method is attributed to its portability, objectivity, minimal invasiveness, high accuracy and good precision. Although a relatively standard protocol for the method has emerged, the new generation of users often is unfamiliar with rationale behind aspects of the protocol as well as the approaches to avoid or correct for in situations that are not covered by the standard protocol procedure. The primary uncommon situations like introduction of isotopically different diet and fluids with or without geographical relocation, seasonal and temperature variations, activity level of participants etc. during or prior to the DLW measurements can lead to shift in baseline abundance of ^2^H and ^18^O or tracer elimination, resulting in moderate to large errors in the measured TEE. These unique situations call for special modifications to the conventional protocol to minimize errors. The objective of the present review was to assemble a list of frequently asked questions and the issues they represent, and then examine the available literature to describe and explain the modifications to the standard DLW protocol to maintain the method’s accuracy. This discussion of DLW protocol modification can be an excellent resource for investigators who intend to use this measurement technique for interesting and uncommon study designs.

## Introduction

The estimation of energy expenditure in free-living organisms has been recognized for years to be central to understanding energy balance. One of the most impactful advances in the measurement of energy expenditure was the development of the Doubly Labeled Water (DLW) method by Lifson and McClintock, which was first validated in animals in 1955 [1]. It was not until twenty-five years later, however, that Schoeller and Van Santen successfully applied this method in humans [2]. Given its importance in the field of bioenergetics, it is not surprising that this technique is still considered the gold standard method to assess free-living total daily energy expenditure and has been validated for use in all populations including pregnant women, infants and elderly.

If all assumptions upon which this method relies are satisfied, human energy expenditure can be measured with 2% accuracy and 2–10% precision using a standard DLW protocol [3]. The cost of enriched ^18^O labeled water, however, requires that loading doses of doubly labeled water be small. Because both ^2^H and ^18^O are naturally occurring, these small doses result in the natural abundances of these stable isotopes being mathematically important in the calculation of enrichments following the loading dose. Under certain study conditions, changes in physiological or environmental variables can introduce a measureable shift in the natural abundances producing errors in the calculated enrichment and hence Total Energy Expenditure (TEE). Thus, the main purpose of the present review is to (i) summarize the rationale for the DLW standard protocol, (ii) identify those unusual study situations where the accuracy of the doubly labeled water method would be impacted and (iii) discuss approaches and options to avoid or correct for such variations.

## Measurement of TEE in Humans Using DLW

The method involves loading the body with water labeled with non-radioactive stable isotopes of hydrogen (^2^H) and oxygen (^18^O). This raises or enriches body water relative to the natural levels of ^2^H and ^18^O, allowing for the measurement of total body water by the dilution principle. Overtime, the concentration of these isotopes decreases (5–20% per day) in the body due to consumption of non-enriched water from foods and fluids, which is accompanied by loss of isotopically enriched body fluids such as urine and sweat, and evaporation from lungs and skin. The ^2^H is lost almost exclusively as water, while ^18^O is lost almost exclusively as water and C. The differences in the elimination rate calculated from a decrease in the tracer abundances in body water along with the amount of total body water is then used for calculating the CO_2_ production. The latter along with an estimate of the respiratory exchange ratio is used to calculate TEE.

### Calculating the TEE from the urine

The elimination rates of ^2^H (k_H_) and ^18^O (k_O_) are commonly calculated using the two point sampling method [4–6] and occasionally multiple points sampling method [3,7]. Changes in the natural log of isotopic enrichment as a function of time elapsed after dose administration is calculated for both deuterium (^2^H) and ^18^O. The mean enrichment in urine samples collected on the day of dosing and day 14, with the number of days elapsed are used in calculation of elimination rates. To a first approximation, the CO_2_ production rate is then estimated as:

rCO_2_=(TBW/2)(k_O_ − k_H_)

This, however, does not account for isotope fractionation or differences in isotope distribution spaces. Doing so for humans greater than 1 year of age, leads to a more complex equation [8]:

rCO_2_=(1/2f_3_)(N_O_k_O_ − N_H_k_H_)−r_G_(f_2_ − f_1_)/2f_3_

Where: f is the fractionation factor for the conversion of 1) hydrogen isotopes in water vapor and liquid water, 2) oxygen isotopes in water vapor and liquid water, and 3) oxygen isotopes in CO_2_ and liquid water; N_O_ and N_H_ are the dilution spaces for labeled oxygen and hydrogen, respectively; k_O_ and k_H_ are the disappearance rates for labeled oxygen and hydrogen, respectively; and r_G_ is the rate of fractionated water lost as gas (transcutaneous and lung).

Substituting for the known constants, this simplifies to [5,8]:

rCO_2_=0.455 TBW (1.007 k_O_−1.041 k_H_)

Where, TBW=Total Body Water in moles; k_O_ and k_H_=oxygen and deuterium elimination rates in pools/day.

### Calculation of elimination rates

The elimination rates of oxygen (k_O_) and deuterium (k_H_) are calculated by the two point method using the initial and final enrichment [4]. Collection times are converted into decimal notation in days and plotted against log enrichment values to calculate k_O_ and k_H_:

k=−(ln enrichment_final_−ln enrichment_initial_)/Δt

Where, ln=natural log; enrichment=enrichment above baseline; Δt=enrichment between the initial and final sample.

### Calculation of TBW

The TBW is calculated by urine analysis from the equation developed by Cole and Coward (7):

Isotopic dilution space=((WA/1000a)×(da−dt)/(ds−dp))−w

Where, W=quantity of water used to make isotopic dilution in grams; a=quantity of dose water used in dilution in grams; A=quantity of dose taken by participant in grams; d_a_=the permil isotopic enrichment measured in the diluted dose; d_t_=the permil isotopic enrichment measured in tap water used for dilution; d_s_−d_p_=enrichment measured in post-dose urine minus pre-dose urine sample; w=amount of water consumed between the DLW dose and 3 hour post dose urine sample.

The TBW is calculated from the average of ^2^H and ^18^O dilution space divided by 1.041 and 1.007 respectively to correct for in vivo isotopic exchange [5].

### Calculation of TEE

From rCO_2_ the TEE is calculated using modified Wier equation [9]:

TEE(kcal/day)=22.4(3.9(rCO_2_/FQ)+1.1(rCO_2_))×4.184/1000

Where the Food Quotient (FQ) is substituted for the 24 h Respiratory Quotient (RQ), or more correctly Respiratory Exchange Ratio (RER=CO_2_ produced/O_2_ consumed). Modification may be required to account for use of storage of energy substrates, if the subject is 20% or more outside of energy balance [10].

These calculations are used to accurately measure both the total daily energy expenditure as well as body composition based on the following assumptions [3]:

1. The volume of the body water pool is constant (+/−3%) throughout the measurement.
2. With the exception of known isotopic fraction between water, water vapor and CO_2_, the concentrations of isotopes leaving the body are same as those in body water.
3. The isotopes label only H_2_O and CO_2_ in the body and the rapidly exchangeable H and O that equal 4.2% and 0.7% of the pool, respectively.
4. None of the two isotopic tracers that leave the body re-enters.
5. The natural abundance of both isotopes remains constant during the measurement period.
6. The average respiratory exchange ratio is equal to the food quotient for the duration of the measurement.

## Standard Protocol for DLW Administration

### DLW administration and sample collection

The dose for the DLW is calculated for each individual with the ratio of 0.09 to 0.12 g ^2^H_2_/kg TBW and 0.18 to 0.23 g H_2_^18^O/kg TBW [11,12]. The dose is sterilized by filtering through 0.22μm filtering system and sealed in a sterile 80 to 150 mL bottle. All handling materials for the isotopic water must be free of other sources of water that would contaminate the dose and evaporation should also be avoided. Presence of contamination or evaporative water loss that may alter the dose weight by more than 0.5% will result in measureable and proportional errors in TBW and TEE. Doses can be stored for up to 9 months in a cool and direct-sunlight free area before evaporative loss and loss of sterility become concerns. Refrigeration also reduces any minor unpleasant smell and taste of the water and is better tolerated by the participants.

The most commonly used DLW protocol is the modified “two point” plateau method [7]. On the day of the DLW administration a baseline urine sample is collected to determine the enrichment and to ensure that subjects have voided before the DLW dose, followed by an overnight fasting. After the DLW oral ingestion, participants consume a small quantity (50–100 ml) of drinking water to wash out dose bottle and mouth to avoid incomplete dosing and loss of highly enriched water by evaporation from the oral cavity. Time of administration, duration of dosing and any spillage are recorded. Three additional urine samples (>20 ml to minimize artifacts due to evaporation or contamination with ambient water vapor) are collected, with the first at least 1 hour and the last two at least 3 hours post DLW dose administration [13]. These samples are then sealed and frozen until the analysis is performed. Two final urine samples are collected at the end of the period of TEE measurement, typically between 4 to 21 days depending on the turnover of the two isotopes [4]. Samples should be collected on the same time of the day (+/− 3 h) to minimize small errors due to diurnal variations in energy expenditure and water flux. Time of sample collection should also be noted correctly to make adjustments in the calculation of elimination rates. Because the time of voiding is not equal to the time the urine entered the bladder, it may add an error in the TEE measurement. However, this error is small because it occurs for every urine specimen. It is very valuable to collect and store backup samples, especially at the beginning and end of the DLW process to avoid problems related to loss or contamination of specimen.

Additional protocols are the “slope-intercept” protocol in which the urine samples are collected beginning 6 to 24 hours after the dose and the initial isotope dilution is calculated by back extrapolation of the ln (enrichment) vs. the time curve [14]. Post dose samples can be collected on day 1 and the final day of the elimination period or as frequently as daily during the elimination period. More frequent sampling can improve precision, especially if the measurement of enrichment is a major source of random error in a given laboratory, but this does increase the analytical workload [7]. Finally, the Maastrict protocol has been developed which uses an evening dosing and an overnight period before collecting the first urine to ensure isotope equilibration [15]. These protocols, although slightly different, provide very similar (+/−1–3%) results and thus decisions on which to use is typically made to best fit the practical aspects of the study.

### Sample isotopic analysis

Isotope Ratio Mass Spectrometry (IRMS) is commonly used to measure the isotopes in the body fluids based on the methods explained in IAEA 2009 report and the OPEN study [3,12]. During the past few years, Cavity Ring-Down (CRD) laser spectroscopy has become a competing analytical technique. This instrumentation has the potential to be faster and less expensive, but at this time does suffer from memory or carry-over between successive specimens that creates a trade-off between accuracy and speed [16,17].

## Frequently Asked Questions Regarding the Standard Protocol

### Should food and fluid intake be restricted on the day of DLW administration?

In most studies participants are not allowed to consume food and water during the 3–6 hours of equilibration period to prevent expansion of dilution space. Lack of food and water, however, can also result in discomfort and difficulty in producing urine specimens. Therefore, it is now recommended to consume a small meal (<15% of daily energy requirement) or beverages (<500 mL) between 1 and 3 hours after DLW administration and then subtract the volume of that food and beverage from the calculated TBW.

In special populations such as infants where the equilibration period exceeds the recommended feeding schedule, food can be consumed with the dose or as soon as 0.5 hour post dose. In this instance, investigators have generally used the slope-intercept protocol because it allows calculation of the enrichment at the time of dose [14]. Among older subjects, food and beverage consumption is delayed to 1 h after dose to allow for gastric emptying of the dose, an approach that can be used with the plateau protocol [18]. Once consumed, any extra food moisture or water equilibrates with body water and therefore increases the apparent dilution space [2]. Subtracting the extra fluid intake from the isotope dilution space corrects the TBW value back to value at the time of dosing [18]. Trabulsi et al., for example, conducted a study in 484 subjects to evaluate the precision of the DLW technique for a large-scale evaluation of dietary intake instruments [12]. The researchers allowed 600 mL of liquid replacement meal (Boost, Mead Johnson Nutritionals, Evansville, IN, USA), coffee, tea, juice, or water between 1 and 3 hours of DLW. The time, type and quantity of beverage consumption was recorded to correct future TBW calculations [19]. Liquid consumption should not be allowed between 3 and 4-hour post dose unless the participant is unable to void due to slow urine production [12]. Amount of water larger than 600 mL approach quantities that have been shown to cause temporary isotopic disequilibria in the circulatory water space resulting in overestimation of the dilution spaces [20].

### Why are the urine samples collected at specific time periods?

The urine sample collected at baseline is used to measure the baseline natural isotope enrichment levels in the body. A DLW dose is administered and specimens are collected again at 1 or 2, 3 and 4–6 hour time point to determine the urinary isotopic enrichment above the baseline levels. The urine specimens collected after the dose administration, but before 3 hour post-dose clear the bladder of water produced before isotopic equilibration and are usually not analyzed [13]. The urine specimens collected after 3 hour, if equilibrated, will have enrichments that agree within 5% and are used for calculating TBW. A disagreement of more than 5%, however, will indicate failure to reach equilibrium [7]. The equilibrated specimens collected after the dose along with the isotopic enrichment of urine sample collected at the end of the metabolic period are used to calculate the elimination rates. The pair of specimens collected over a short time interval provide for replication to reduce errors in calculating TEE. It takes longer for the isotopes to reach equilibration in pregnant women; therefore the urine should be collected 4 hour post dose administration as compared to 3 hour in non-pregnant women [21].

### What if the participant cannot produce a urine specimen at the designated time?

Efforts can be made to induce urine production by techniques like letting the participant hear water running, inducing relaxation by placing their hand in warm water, or increase urine production by placing their hand in cold water. Allowing consumption of another 250 mL of water may also help with urine production. If the participant still cannot produce a urine specimen at the designated time, sample collection can be delayed until urine specimen is produced and the actual collection time and date should be recorded.

### Why is the elimination period two weeks?

The optimal observation period for the two isotopes ^2^H_2_O and H_2_^18^O is between 1 and 3 biological half-lives of the ^2^H isotope, which varies from 3 days in infants or endurance and other athletes to 2 and 3 weeks in sedentary elderly subjects [22]. Deuterium is eliminated from the body as water and the ^18^O as water and CO_2_ and the difference between the disappearance rates of these two isotopes gives us a value for CO_2_ production.

### How can TBW be used to measure body composition?

An important feature of the DLW method is its ability to measure body composition in humans [23]. Because both isotopic tracers distribute quickly in body water with reproducible exchange into non-aqueous compartments, body composition can be measured at the same time as TEE measurement. The principle for measurement of TBW is the dilution principle in which the products of pool size, tracer enrichment in the dose, and body water are equal. Thus, when the dose mass and isotope enrichment of the dose is known, the mass of the isotope dilution space can be calculated from the enrichment of isotope in body water at equilibrium as indicated by the equation shown above. For humans greater than 1 year of age, the TBW can then be calculated from either or both the isotope dilution spaces where [5]:

TBW=N_H_/1.042=N_O_/1.007

During the first year of life, these calculations may differ, suggesting that the calculation of TBW may be less prone to error [24,25].

Calculating Fat Mass (FM) and Fat Free Mass (FFM): FFM is derived from dividing the calculated TBW with the hydration coefficient of 0.732 in adults [26]. The FM can be calculated by subtracting derived FFM from body weight.

FFM(kg)=TBW/0.732
FM(kg)=body weight (kg)−FFM (kg)

The hydration values for infants, children, and adolescents vary by age [27,28].

### Can DLW method be combined with the other stable isotope labeled substances?

Some metabolic study designs call for tracers other than ^2^H and ^18^O for metabolic outcomes and may involve administering multiple stable isotope labeled substances at the same time. Isotopic tracers, such as ^13^C, do not interfere with the IRMS or the CRD analysis, as shown by both animal [29,30] and human studies [31,32]. Caution must be exercised, however, with compounds labeled with ^2^H and ^18^O as these may interfere with the analysis if they are metabolized to or exchange with water.

### Can DLW method be used for large epidemiological studies?

A study has successfully validated the use of DLW method in measurement of energy expenditure in a large group of 484 adults [12]. A major issue with using the DLW technique in epidemiological studies is delays involved in weighing the dose water during clinic time. To alleviate this issue, researchers prepared five different dose weights of DLW prior to the start of the study. The dose weights were calculated based on the TBW measurement for gender-dependent median body weight and TBW calculated from adult bioelectrical impedance analysis data from the National Health and Nutrition Examination Survey. The study confirmed that the DLW maintains its precision in a large-scale setting with a streamlined-dosing protocol [12]. The costs of ^18^O and isotopic analyses are high and typically limit the measure to a subsample of the cohort. Further improvements in cavity ring-down spectroscopy are expected to reduce the analysis cost.

## Potential Artifacts when using DLW Method under Unusual Conditions

The DLW technique measures energy expenditure in free-living humans with a proven accuracy of 2% and a precision of 2–10% [4]. For this measure to be accurate however, the isotopic abundances of water entering and leaving the body, with the exception of the loading dose, need to be at steady state [33]. This requires accounting for interventions that involve changes in environmental or physiological factors like physical activity, health status, that alter isotopic background [34] or isotopic fractionation [35]. It may not be feasible to control all these variables, but carefully manipulating either the dose of DLW provided or the duration of metabolic period can avoid under or over estimation of the TEE data obtained [7,11]. Given the broad prospects for application of this method for measuring energy expenditure, the following studies provide evidence and direction for researchers who wish to use this technique in similar research situations.

### Influence of diet/fluid on baseline tracer levels

Typically the isotopic background of body water is assumed stable for the duration of a conventional DLW measurement protocol. Changes in diet pattern or geographic location, however, can alter the natural abundance of ^2^H and ^18^O in body water causing baseline isotope shifts. This variation in the isotopic background results in errors in the calculated body water turnover rates, giving inaccurate information on CO_2_ production [36]. Existing data suggest that different strategies can be employed to accurately measure energy expenditure using DLW in these unique study situations of changing diet/fluid with or without changes in geographic location.

### Change in diet/fluid with no geographical relocation during the dose administration and tracer collection

Certain study situations require an introduction of diet or fluids that are isotopically different from local drinking water, at the beginning of the tracer collection period. Such study conditions include introduction of the Total Parenteral Nutrition (TPN) solution with different natural ^2^H and ^18^O in hospitalized ambulatory patients [37], oral or parenteral diet in infants with abdominal surgery [38] and a change from breast feeding formula or solid foods [39]. In other study designs, a new diet/fluid is introduced midway through the tracer collection period in order to evaluate the shift in energy expenditure with a shift in diet [40]. These different study designs call for different strategies to reduce systematic errors in energy expenditure measurement.

Foods vary in abundances of ^2^H and ^18^O and will change the isotopic background when introduced during the tracer collection period. A prime example is intake of a variety of complementary foods in infants in conjunction with breast-feeding, a situation that creates a unique challenge for the DLW studies that intend to measure energy requirements of infants. Typically, a small dose of isotopic water ranging from 1 g/kg TBW-2.6 g/kg TBW is administered to the infant and urine or saliva samples are collected between 5–15 days and has been extensively used to measure energy intake in infants who are exclusively breast-fed or formula fed [41–46]. When, however, these infants reach an age where complementary foods are introduced during the tracer collection period in conjunction with breast-feeding, the isotopic background changes. Adult studies involving a switch to a special formula diet to study the effects of specific macronutrients is another example [47]. Both are indications for utilizing strategies that can account for unknown changes in food consumption.

#### Correction of baseline tracer levels using an equilibration period

In situations where the dietary change to be introduced during the tracer metabolic measurement period is known in advance, it is advisable to perform an equilibration and wait for the new isotopic baseline of body water to be formed [37]. For example, Schoeller et al. measured the energy requirements of hospitalized ambulatory patients on the Total Parenteral Nutrition (TPN). Since the TPN solution is formulated using distilled or otherwise purified form of water sourced near the formulary and thus having an isotopic natural abundance different from body water, the patients were maintained on a consistent TPN diet for 10 days before administering the DLW dose [37]. This equilibration period reduced fluctuations in the natural abundance of ^2^H and ^18^O in body water due to introduction of a new water source, thus reducing errors in estimating isotopic enrichment at end of the energy expenditure measurement period. Jones et al. also performed a similar, but shorter equilibration of 4 days in infants who were put on either oral or parenteral diet post abdominal surgery [40]. Thus, an equilibration period of approximately one half-life of the body water pool is a viable option for similar study designs.

#### Correction of baseline tracer levels using prediction models

**Figure 1:**
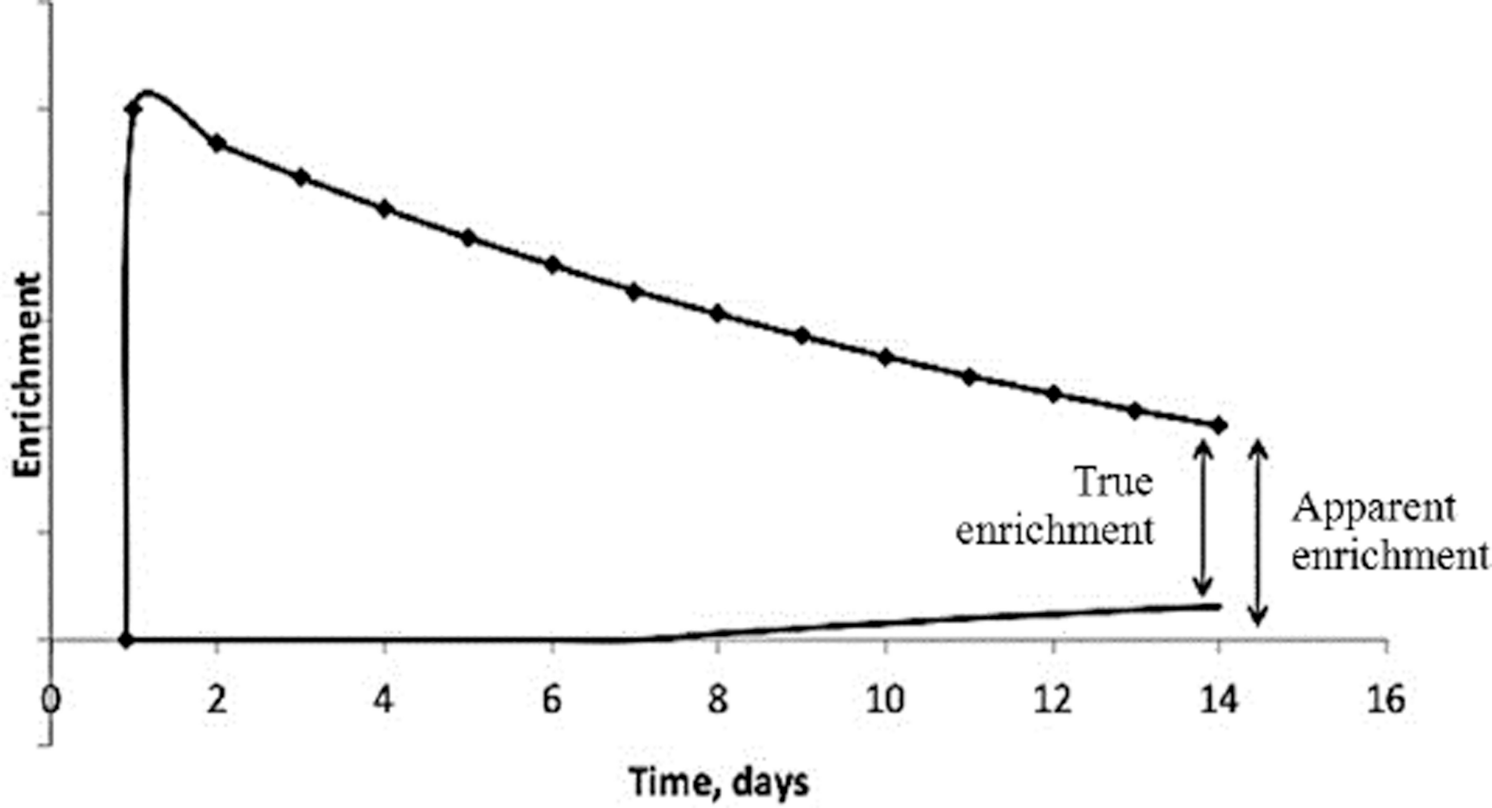
When the baseline changes after the dose of DLW, the change cannot be readily observed (see text). This can cause an error in the calculation of TEE because the apparent enrichment above the predose baseline is no longer the true enrichment.

In cases where shift in the diet is made in the middle of the TEE measurement period, different correction methods are required to adjust to the new baseline. For instance, Jones et al. studied the precision of the DLW to measure energy expenditure in infants postoperatively, where the diet was switched from parenteral to an alternative parenteral/oral formula regimen (shift in the nutrient related water) at the midpoint of the measurement [40]. Conventionally, isotopic enrichment of the body water is measured by differences between the isotopic abundance after the DLW administration and the baseline. When the diet, and especially when the water in the diet changes post DLW dose administration, the baseline changes. That change, however, cannot be readily detected during the TEE period until the loading dose washes out in about 10 half-lives or two to three months (Figure 1). Thus, the pre-dose baseline is not a reliable representation of the true baseline. In similar situations, researchers may use the predictive equations developed by Jones et al. that take into account differences between measured initial baseline and predicted baseline on the new diet, the time interval to reequilibration to new water, and fraction of water derived from dietary source [40]. The body water change was predicted by comparing the final isotopic abundance adjusted for normal enrichment of body fluids relative to intake, with the initial body isotopic abundance.

#### Obviating the error using a high loading dose: Administration of large dose of isotopes can also be an option to reduce measurement errors

Propagation of error calculations demonstrate that baseline isotopic shifts will have a negligible effect on the TEE measurement when the difference between the pre-dose baseline specimen and the predicted shifted baseline is less than 0.7% of the enrichment of the final urine specimen. In this case the error in the TEE will usually be less than 4%. While a useful approach, especially under conditions where there is little control of diet and beverage intake, this approach does increase the cost of the DLW loading dose.

### Change in diet/fluid with geographical relocation during the dose administration and tracer collection

The natural abundance of deuterium and ^18^O varies by the geographical location with ^2^H ranging from 80–180 ppm and ^18^O from 1900 to 2050 ppm. These isotopes are also naturally present in the body’s organic compound. When a person moves to new geographic location and drinks water of a different isotopic abundance, the isotopic enrichment of the total body water changes. This is challenging for those DLW studies where participants are moved to different location during the metabolic measurement period [34]. For example in military or astronaut nutrition studies, participants are commonly transported to a different geographical location for their field assignment, changing the isotope abundance and causing baseline isotopic shifts [48–54]. Following methods can be used to control for errors associated with administering the DLW at different geographic locations.

#### Correction of the baseline isotopic shifts using an equilibration period

Introducing a period of equilibrium where subjects consume water from the new geographic location for 1–3 weeks before baseline dose administration is suggested to avoid underestimation of energy expenditure [37]. This method has been validated [48], but if the rate of turnover is low or if the subjects are traveling to a different location for a short period, this method may not be practical.

#### Correction of the baseline isotopic shifts using a control group

To account for the differences in isotopic proportions of water at a different location, it is also advisable to enroll a control group that does not receive any tracer. It is important that the control group should be involved in similar physical activity as the isotopic group for consistent isotopic elimination rate. The average change in the elimination rate of that control group is then used to estimate a baseline correction for the test group. Three studies used the control group data to correct for the error in raw isotope data [48,49,51]. DeLany et al. tested the effect of change in food and water source on energy expenditure measurement post DLW dosage administration in soldiers during a 30-day field training [48]. The control group did not receive any heavy water. A significant decrease in ^2^H and ^18^O enrichment was observed in urine samples in soldiers receiving no loading dose. These values from control soldiers were used to establish and predict the change in baseline and for calculation of the enrichment [48]. Jones et al. also studied the energy expenditure in military troops who moved to the Arctic Circle for 10-day training [51]. Similar to Delany et al., in this study control group showed a shift in the baseline isotopic enrichments by −4650 and −480 ppm per day for ^2^H and ^18^O, respectively, primarily due to low temperatures (−25 degree Celsius) and changes in food and water source. These shifts in the control group were then used to correct for the DLW measures in the experimental group [51]. In another study of energy expenditure measurement, marines were transported to a high elevation region with temperatures of −15 to 30 degree Celsius for 11-days of high intensity training exercise. The DLW was administered to all marines except the controls and corrections were made for the shifts in the isotopic baseline for the experimental group [49]. Therefore, using control subjects maintained the accuracy of DLW.

#### Correction of the baseline isotopic shifts using a combination of equilibration period and control group

Multiple studies measuring energy expenditure during military field missions have combined equilibration period and inclusion of control group to increase accuracy of the method. Gretebeck et al. employed both these correction methods for errors due to background isotope change, where subjects were to consume water from a given source indefinitely [53]. Eight healthy women consumed tap water enriched with ^2^H and ^18^O to resemble the water available on a typical space shuttle mission for 28-days. The same enriched water was used to prepare drinks and rehydrate food as well. Additionally, a control group was also included in the study that received the enriched water for 14-days for equilibration and given DLW on day 15. When the isotope disappearance rate was calculated without considering change in the background, an error of 2.9 MJ/day in TEE was observed in the 28-day enriched water consumption group [53]. This study validated the use of an equilibration period of minimum 2 weeks to the new water source, when the isotopic enrichment of the water source for subjects is altered. The study also confirmed the validity of a control group to track changes in the isotopic background. In cases where control group is not feasible due to limited number of subjects, baseline changes can be corrected based on the final isotope ratio of the fully equilibrated baseline isotope abundance [53]. This research group took a similar approach in another study where energy requirements were calculated for 13 healthy males during a ground based mission (control) or a space flight for 8–14 days. Levels of deuterium and ^18^O in the potable water for space mission are quite different from that of ground water and therefore a modified DLW method was used to compare the two groups. The enrichment levels of potable water were predicted from the hydrogen and oxygen gases used for the fuel cells and a preload of deuterium and ^18^O were given to each crewmember to raise their baseline isotope levels to that of potable water. Dosing before the flight changed the isotopic levels of the astronauts and reduced the differences between the isotopic abundances in preflight body water and drinking water in the flight [54]. The modified combination approach has also been successfully used in a similar study where energy expenditure was measured in soldiers during a high intensity field exercise at high altitude of Mt. Rainier by obtaining a supply of the drinking water from the destination local. Soldiers drank water from the ranger station at Mt. Rainier 10-days prior to the DLW administration bringing the body of the subjects in equilibrium with the water isotope levels in the new location. The study also employed a control group to correct for the values [50]. Similarly, the African Bush soldiers were put on a 1-week equilibration diet before high intensity field training in combination with a control group to avoid errors due to changes in the baseline body water isotopic abundances [52]. The combination method has been the most popular method to correct for baseline shifts.

#### Correction of the baseline isotopic shifts using a high isotopic dose

Corrections for baseline isotopic shifts can also be made by simply increasing the dose of the isotopes above the level of the baseline in the natural environment [37]. A larger dose increases the signal relative to the natural abundance of deuterium and ^18^O and to the random error in the isotopic measurement, thus improving the precision [53], but costs for the labeled water also increase.

#### Correction of the baseline isotopic shifts using a loading dose that mimics the natural isotopic abundance ratio

^2^H and ^18^O have a covariant relationship, which means any shifts in the natural abundance of both isotopes will be in the same direction and proportional. Therefore, to dramatically reduce any errors due to change in the natural isotopic abundance with change in geographic location, it is recommended to use a loading dose that mimics the ratio of natural abundances across the world [34]. In doing so the change in baselines for the two isotopes will introduce errors into the isotope elimination rates, but those errors will cancel each other when the difference between k_D_ and k_O_ is taken while calculating TEE.

#### Delaying the loading dose

Baseline isotopic errors can occur not only when a person is transported to a remote study site, but also when a person travels large distances weeks before or during the DLW period and then returns to the dosing site. For example, if a person has traveled more than 200 miles or traveled shorter distances that involve large changes in altitude two weeks before or plans to travel during the DLW period, the baseline isotopic abundance may change [34]. To avoid errors due to changes in isotopic abundance with travel, it is recommended to delay the DLW dosing for at least one biological half-life.

### Influence of seasons or ambient temperature on baseline tracer levels

Change in seasons affects isotopic levels by 1) changing the isotopic abundances of the main water source, or 2) by changing the water turnover rate in the body. Seasonal variations in drinking water were noted by Riumallo et al., who conducted DLW administration in two phases in subjects living in Santiago, Chile [55]. Urine samples were collected 12 weeks after the first DLW phase followed by new dose administration. Complete elimination of excess isotopes was expected after 12 weeks, but surprisingly both baseline deuterium and ^18^O abundance increased after this period, which may have been influenced by reduced contributions of snow melt to the water source [55]. The seasonal affect on isotopes has also been observed in tropical regions or during summer season when major changes occur in the sources of drinking water, atmospheric water vapor, and evaporative water loss rates from the body [56,57]. More recently, Harbinson et al. determined that both ^2^H and ^18^O were more enriched in urine samples during the dry season compared to the rainy season, when measured over 12 months in Nigeria [58]. The abundance of these isotopes was positively associated with mean monthly temperature and was inversely associated with rainfall and minimum relative humidity [58]. These results imply that the error due to high water turnover and changes in the fraction of water lost through evaporative processes in high temperature and humidity conditions is inevitable, but appropriate adjustment and/or control of the ratio of ^2^H and ^18^O in dose water will avoid measurement errors and improve accuracy [49,55].

#### Correction of errors due to seasonal variations

One way to account for errors in isotopic elimination measurement is by applying theoretical corrections for the environmental water intake via skin and lungs. For instance, Fjeld et al. measured the intake of breast milk in infants using the deuterium dose under high temperature and humidity conditions [59,60]. High water content of atmosphere and subsequent reduction in food intake resulted in non-dietary water accounting for 30% of the total water influx and therefore overestimation of milk intake. But when researchers corrected for this atmospheric water influx the error was reduced [59]. To deal with the seasonal issue, Luke et al. employed another approach in a study where energy expenditure was measured in 149 women in tropical conditions of Nigeria [61]. Due to a potential rapid body water turnover with high heat and humidity, the researchers reduced the isotope elimination measurement period in the study to 7–10 days from conventional 14-days improving the accuracy of the measurement. This resulted in higher enrichments of the tracers in the final specimens and thus reduced the influence of baseline changes.

### Influence of high activity on tracer elimination levels

The DLW method has been employed in study situations with high activity levels including studies on soldiers on high intensity training, wildlife firefighters, professional athletes and amateur athletes [48–52,62,63]. These high physical activity situations create two potential sources of error for the DLW method. The first is that at high volumes of physical activity, water loss as sweat and breath vapor increases resulting in rapid water turnover. With rapid water turnover the portion of water lost through isotopically fractionation routes also changes [35,48] and should be accounted for when using the labeled water. A second source of error is a change in the natural abundance of ^2^H and ^18^O in body water with changing geographic location, as reported for soldiers and marines during high activity field exercises [48–50]. These changing environmental conditions, in addition to the physical activity levels further shift the water turnover rates to a higher level. For instance, the r^2^H_2_O in the wildfire fighters was reported to be 6.7 ± 1.4 L/24hr as compared to recreationally active college students (3.8 ± 1 L/24hr), possibly due to a combination of high heat and high physical activity [62]. Similarly the water turnover significantly increased from 45 ± 7 ml/kg/day to 60 ± 10 ml/kg/day during the ascent to a high altitude of 8,047 m in 13 healthy adults [63]. To account for this rapid water turnover with high physical workload two approaches in modifying the DLW method are recommended.

#### Correction of errors by reducing the length of observation

The length of metabolic measurement period can be reduced in high activity study situations to avoid loss of precision when the final isotope enrichments become small [9]. It is important to determine the intensity of the physical activity in advance because the half-life of isotopes decreases with increased water flux and fractionation due to heavy activity. For example, Westerterp et al. administered the DLW at an interval of 7, 8 and 7 days, and recorded the isotopic elimination rates during a 3-week period of high intensity bicycle race [64]. The half-lives of isotopes were reported to be one third of the usual values in humans with normal activity levels. The short half lives were a result of high energy expenditure and researchers recommended reducing the length of the metabolic period to 2–4 days for maximum precision [64]. The same research group provided more evidence in this area by comparing the isotopic elimination rates in low activity group with a high physical activity group [22]. The daily elimination rate of ^18^O and ^2^H were two fold higher in the high activity group than the low activity group resulting in a short biological life of 4–6 days in the high activity group vs 6–10 days for low activity levels [22].

#### Correction of errors by correcting for fractionation

The above extreme conditions may also influence the fractionation corrections that are incorporated into the calculation of rCO_2_. This is because neither sweat nor urine is subject to isotope fractionation, while breath water vapor and transcutaneous water loss are [35]. Thus the proportion of water lost via fractionating routes can change during conditions that dramatically increase sweating or increase if CO_2_ production is increased in the absence of sweating. The potential errors are generally small (<5%), but can be minimized by using the shortened form of equation presented above which does not make an assumption based on the ratio of fractionated vs non-fractionated water loss, but rather assumes the fractioned water fluxes will be proportional to rCO_2_. This theoretical construct has not been tested, however. A triply labeled water method has also been proposed for correction of evaporative water loss increasing the accuracy of heavy water technique [65]. Other published equations that assume a fixed proportion will lead to small errors under these extreme conditions. To account for the errors due to isotopic fractionation of water lost on breath and transcutaneous loss (non-sweat), corrections proposed by Schoeller et al. and Fjeld et al. [8,59] were especially used by studies in soldiers and athletes [48–50,66].

### Influence of repeated DLW before the tracers have been eliminated from the body

**Figure 2:**
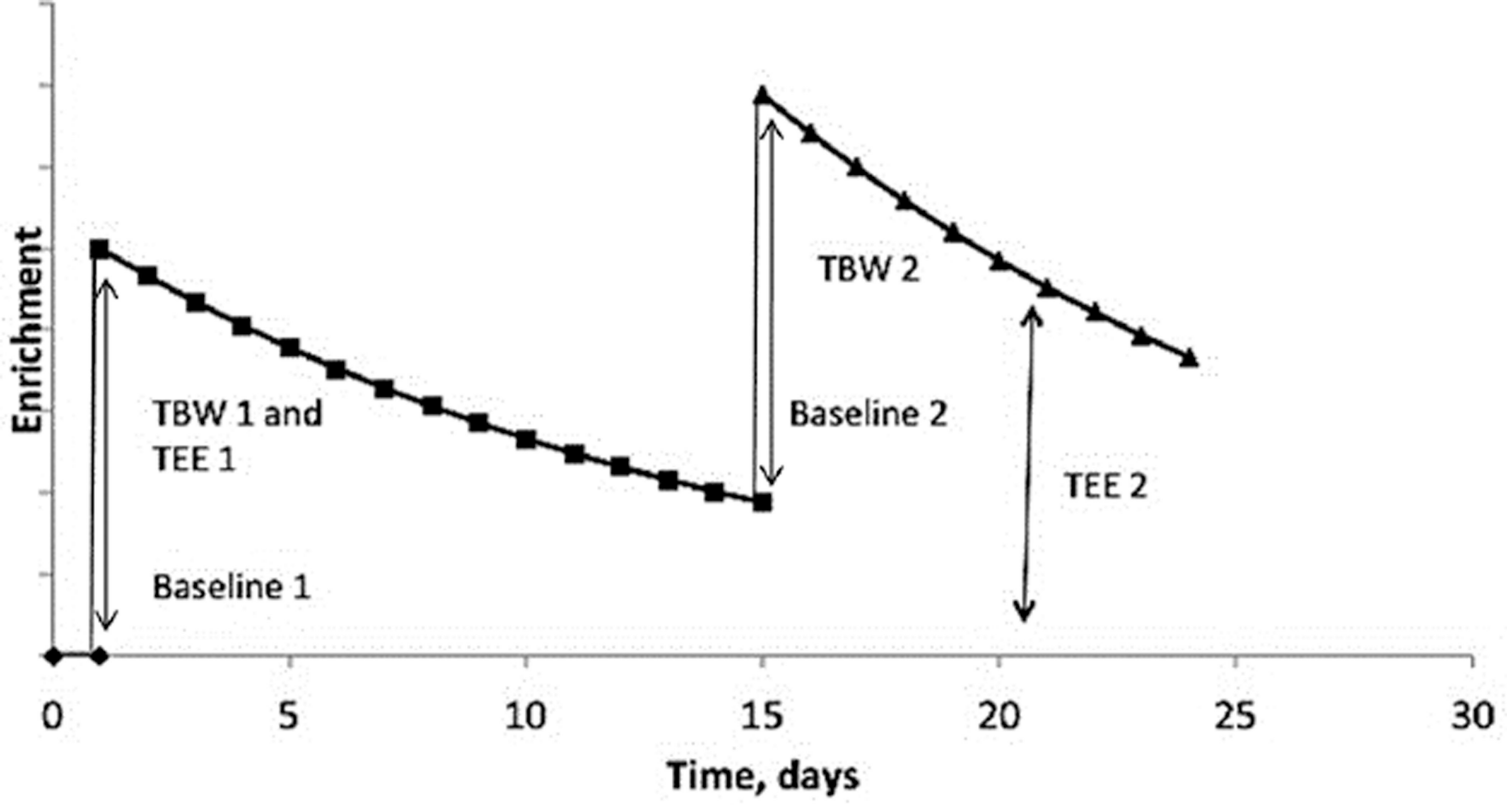
When a second dose is given before the enrichments return to the predose 1 baseline, the predose 1 baseline is used to calculate TBW 1 and TEE 1. The baseline 2 is used to calculate TBW 2, but baseline 1 is still used to calculate TEE 2.

Certain study designs require an administration of a second dose of the labeled water before the tracers from the first dose are completely eliminated from the body. Trabulsi et al. validated the use of repeated TEE measurement in a subset of subjects of a large epidemiological study [12]. The subjects returned to the study site 11–14 days after the first DLW dose to complete the DLW protocol. During that visit a second dose of the labeled water was administered to determine between- and within- person variation in TEE. The isotopic abundance of the body water was still enriched at the time of the second dose. That enriched value was used for calculating TBW, but the original baseline urine collected before the first dose was used to calculate isotope elimination for calculation of TEE for the second dose of the isotopic water [12]. Thus, in studies where a continuous measure of the TEE is required, repeated dose of the DLW may be administered but one must use the local baseline for calculation of TBW and the natural abundance baseline before any dosing for the TEE (Figure 2).

## Summary

Strong evidence that the doubly labeled water technique has broad applicability is presented here and in other reviews [67–69]. Available research demonstrates its successful use in assessment of energy expenditure in variety of participants from premature infants to elderly, healthy to morbidly obese and hospitalized patients to highly active athletes and soldiers.

The technique has also provided accurate data in studies with extreme environmental variations in temperature, altitude and humidity levels, but only when investigators make modest changes in the standard DLW protocol to minimize error secondary to baseline changes or water flux changes. The most common inaccuracies have been attributed to baseline shifts in isotopic abundance of the body, water flux and isotopic fractionation. But with careful manipulation of certain aspects of this method such as modifying isotopic dose, adjusting the length of metabolic period, correcting for fractionation and water flux and correcting for errors using information from a control group, maintaining the isotopic ratio of the dose, those inaccuracies disappeared. Therefore to avoid misuse of the technique, it is recommended that investigators should understand the basic assumptions of DLW technique, and acknowledge that the standard protocol may not be appropriate for all research designs. The need for accuracy becomes even more important when we consider the high cost of procedure and difficulties in repetition of the measurement. Hence, with careful pre-planning and slight adjustments in the protocol, the DLW technique is still considered the most accurate and a “gold standard” method for understanding aspects of energy balance.
